# Supplementary material for: End Sequence Analysis Toolkit (ESAT) expands the extractable information from single-cell RNA-seq data
Source: Genome Res. 2016 Oct;26(10):1397–410. doi: 10.1101/gr.207902.116 (PMC5052061; doi:10.1101/gr.207902.116)
Supplement: Supplemental Material [file supp_gr.207902.116_Supp_Materials.tar.gz › ESAT-esat_v0.1/doc/html/classbroad_1_1core_1_1annotation_1_1_annotation_reader_3_01_t_01extends_01_genomic_annotation_01_4-members.html]

ESAT: Member List


ESAT


- Main Page
- Related Pages
- Packages
- Classes
- Files

- Class List
- Class Index
- Class Hierarchy
- Class Members

All Classes Namespaces Files Functions Variables Enumerator Pages

- broad
- core
- annotation
- AnnotationReader< T extends GenomicAnnotation >

broad.core.annotation.AnnotationReader< T extends GenomicAnnotation > Member List

This is the complete list of members for broad.core.annotation.AnnotationReader< T extends GenomicAnnotation >, including all inherited members.

|  |  |  |
| --- | --- | --- |
| addAnnotation(T annotation, int track) | broad.core.annotation.AnnotationReader< T extends GenomicAnnotation > |  |
| addAnnotation(T annotation) | broad.core.annotation.AnnotationReader< T extends GenomicAnnotation > |  |
| addAnnotationSet(AnnotationSet< T > track) | broad.core.annotation.AnnotationReader< T extends GenomicAnnotation > | protected |
| addAnnotationToLastTrack(T annotation) | broad.core.annotation.AnnotationReader< T extends GenomicAnnotation > |  |
| addBrowserLine(String line) | broad.core.annotation.AnnotationReader< T extends GenomicAnnotation > |  |
| addFilter(Predicate< T > filter) | broad.core.annotation.AnnotationReader< T extends GenomicAnnotation > |  |
| addFilters(Collection< Predicate< T >> filters) | broad.core.annotation.AnnotationReader< T extends GenomicAnnotation > |  |
| AnnotationReader() | broad.core.annotation.AnnotationReader< T extends GenomicAnnotation > |  |
| AnnotationReader(Collection< T > annotations) | broad.core.annotation.AnnotationReader< T extends GenomicAnnotation > |  |
| clear() | broad.core.annotation.AnnotationReader< T extends GenomicAnnotation > |  |
| cloneRegions() | broad.core.annotation.AnnotationReader< T extends GenomicAnnotation > |  |
| concatAnnotationSet(int setNumber, List<?extends GenomicAnnotation > list) | broad.core.annotation.AnnotationReader< T extends GenomicAnnotation > |  |
| containsAnnotation(int setNumber, String name) | broad.core.annotation.AnnotationReader< T extends GenomicAnnotation > |  |
| containsAnnotation(String name) | broad.core.annotation.AnnotationReader< T extends GenomicAnnotation > |  |
| createAnnotation(GenomicAnnotation a) | broad.core.annotation.AnnotationReader< T extends GenomicAnnotation > |  |
| filterByOverlap(List<?extends Annotation > annotations) | broad.core.annotation.AnnotationReader< T extends GenomicAnnotation > |  |
| filterByOverlap(List<?extends Annotation > annotations, int extensionFactor) | broad.core.annotation.AnnotationReader< T extends GenomicAnnotation > |  |
| filterByOverlapAndOrientation(List<?extends GenomicAnnotation > annotations) | broad.core.annotation.AnnotationReader< T extends GenomicAnnotation > |  |
| filterByOverlapOfLastExon(List<?extends GenomicAnnotation > annotations, int extensionFactor) | broad.core.annotation.AnnotationReader< T extends GenomicAnnotation > |  |
| filterByOverlapOfLastExonOrAfterLastExon(List<?extends GenomicAnnotation > annotations, int extensionFactor) | broad.core.annotation.AnnotationReader< T extends GenomicAnnotation > |  |
| filterByScore(double set1FilterScore) | broad.core.annotation.AnnotationReader< T extends GenomicAnnotation > |  |
| findClosest(final LightweightGenomicAnnotation annotation) | broad.core.annotation.AnnotationReader< T extends GenomicAnnotation > |  |
| findClosest(final LightweightGenomicAnnotation annotation, boolean nonOverlapping) | broad.core.annotation.AnnotationReader< T extends GenomicAnnotation > |  |
| findClosestForAll(final AnnotationReader<?extends GenomicAnnotation > otherReader, boolean nonOverlapping) | broad.core.annotation.AnnotationReader< T extends GenomicAnnotation > |  |
| findClosestForAll(final AnnotationReader<?extends GenomicAnnotation > otherReader) | broad.core.annotation.AnnotationReader< T extends GenomicAnnotation > |  |
| findClosestNonOverlapping(final LightweightGenomicAnnotation annotation) | broad.core.annotation.AnnotationReader< T extends GenomicAnnotation > |  |
| findClosestNonoverlappingForAll(final AnnotationReader<?extends GenomicAnnotation > otherReader) | broad.core.annotation.AnnotationReader< T extends GenomicAnnotation > |  |
| getAnnotation(int setNumber, String name) | broad.core.annotation.AnnotationReader< T extends GenomicAnnotation > |  |
| getAnnotation(String name) | broad.core.annotation.AnnotationReader< T extends GenomicAnnotation > |  |
| getAnnotationList(int setNumber) | broad.core.annotation.AnnotationReader< T extends GenomicAnnotation > |  |
| getAnnotationList() | broad.core.annotation.AnnotationReader< T extends GenomicAnnotation > |  |
| getAnnotationsForSequence(String sequenceName) | broad.core.annotation.AnnotationReader< T extends GenomicAnnotation > |  |
| getAnnotationsForSequence(String sequenceName, int setNumber) | broad.core.annotation.AnnotationReader< T extends GenomicAnnotation > |  |
| getAverageScore(LightweightGenomicAnnotation region) | broad.core.annotation.AnnotationReader< T extends GenomicAnnotation > |  |
| getBaseCoverage(List< T > annotations) | broad.core.annotation.AnnotationReader< T extends GenomicAnnotation > |  |
| getBaseCoverage() | broad.core.annotation.AnnotationReader< T extends GenomicAnnotation > |  |
| getBasesCovered(Annotation region) | broad.core.annotation.AnnotationReader< T extends GenomicAnnotation > |  |
| getBasesCovered(Annotation region, boolean fullyContained) | broad.core.annotation.AnnotationReader< T extends GenomicAnnotation > |  |
| getBasesCovered(Annotation region, int fudgeFactor) | broad.core.annotation.AnnotationReader< T extends GenomicAnnotation > |  |
| getBasesCovered(Annotation region, int fudgeFactor, boolean fullyContained) | broad.core.annotation.AnnotationReader< T extends GenomicAnnotation > |  |
| getChromosomeAnnotationMap() | broad.core.annotation.AnnotationReader< T extends GenomicAnnotation > |  |
| getChromosomeAnnotationMap(int i) | broad.core.annotation.AnnotationReader< T extends GenomicAnnotation > |  |
| getChromosomeBEDs(String chr) | broad.core.annotation.AnnotationReader< T extends GenomicAnnotation > |  |
| getChromosomeBEDs(String chr, int i) | broad.core.annotation.AnnotationReader< T extends GenomicAnnotation > |  |
| getChromosomeIterator() | broad.core.annotation.AnnotationReader< T extends GenomicAnnotation > |  |
| getChromosomeTree(String chr, int i) | broad.core.annotation.AnnotationReader< T extends GenomicAnnotation > |  |
| getChromosomeTree(String chr) | broad.core.annotation.AnnotationReader< T extends GenomicAnnotation > |  |
| getChromosomeTreeMap(int i) | broad.core.annotation.AnnotationReader< T extends GenomicAnnotation > |  |
| getChromosomeTreeMap() | broad.core.annotation.AnnotationReader< T extends GenomicAnnotation > |  |
| getDistancesToClosest(final AnnotationReader<?extends GenomicAnnotation > otherReader) | broad.core.annotation.AnnotationReader< T extends GenomicAnnotation > |  |
| getDistanceToClosest(final LightweightGenomicAnnotation annotation) | broad.core.annotation.AnnotationReader< T extends GenomicAnnotation > |  |
| getLastExons(List<?extends Annotation > annotations) | broad.core.annotation.AnnotationReader< T extends GenomicAnnotation > | static |
| getOverlappers(Annotation a) | broad.core.annotation.AnnotationReader< T extends GenomicAnnotation > |  |
| getOverlappers(List<?extends Annotation > annotationList) | broad.core.annotation.AnnotationReader< T extends GenomicAnnotation > |  |
| getOverlappers(List<?extends Annotation > annotationList, boolean fullyContained) | broad.core.annotation.AnnotationReader< T extends GenomicAnnotation > |  |
| getOverlappers(AnnotationReader<?extends GenomicAnnotation > other) | broad.core.annotation.AnnotationReader< T extends GenomicAnnotation > |  |
| getOverlappers(LightweightGenomicAnnotation annotation) | broad.core.annotation.AnnotationReader< T extends GenomicAnnotation > |  |
| getScoreSum(LightweightGenomicAnnotation region) | broad.core.annotation.AnnotationReader< T extends GenomicAnnotation > |  |
| getSequenceAnnotationMap() | broad.core.annotation.AnnotationReader< T extends GenomicAnnotation > |  |
| getSequenceAnnotationMap(int setNumber) | broad.core.annotation.AnnotationReader< T extends GenomicAnnotation > |  |
| getTrackInfo(int i) | broad.core.annotation.AnnotationReader< T extends GenomicAnnotation > |  |
| IncrementScoreIfOverlap(AnnotationReader<?extends GenomicAnnotation > annotReader, int setNumber, Boolean setToZero) | broad.core.annotation.AnnotationReader< T extends GenomicAnnotation > |  |
| init() | broad.core.annotation.AnnotationReader< T extends GenomicAnnotation > |  |
| intersect(AnnotationReader< T > other) | broad.core.annotation.AnnotationReader< T extends GenomicAnnotation > |  |
| intersect(List<?extends GenomicAnnotation > annotations) | broad.core.annotation.AnnotationReader< T extends GenomicAnnotation > |  |
| load(File source, AnnotationFactory<?extends T > factory) | broad.core.annotation.AnnotationReader< T extends GenomicAnnotation > |  |
| load(BufferedReader br, AnnotationFactory<?extends T > factory) | broad.core.annotation.AnnotationReader< T extends GenomicAnnotation > |  |
| load(File source, AnnotationFactory<?extends T > factory, GenomicAnnotationFilter< T > filter) | broad.core.annotation.AnnotationReader< T extends GenomicAnnotation > |  |
| load(BufferedReader br, AnnotationFactory<?extends T > factory, GenomicAnnotationFilter< T > filter) | broad.core.annotation.AnnotationReader< T extends GenomicAnnotation > |  |
| merge() | broad.core.annotation.AnnotationReader< T extends GenomicAnnotation > |  |
| merge(int setNumber) | broad.core.annotation.AnnotationReader< T extends GenomicAnnotation > |  |
| minus(List<?extends GenomicAnnotation > annotations) | broad.core.annotation.AnnotationReader< T extends GenomicAnnotation > |  |
| numberCovered(List<?extends GenomicAnnotation > annotations) | broad.core.annotation.AnnotationReader< T extends GenomicAnnotation > |  |
| numOfAnnotation(int setNum) | broad.core.annotation.AnnotationReader< T extends GenomicAnnotation > |  |
| parse(String file, GenomicAnnotationFilter< T > filter, AnnotationHandler handler) | broad.core.annotation.AnnotationReader< T extends GenomicAnnotation > |  |
| parse(String file, AnnotationFactory<?extends T > factory, GenomicAnnotationFilter< T > filter, AnnotationHandler handler) | broad.core.annotation.AnnotationReader< T extends GenomicAnnotation > | protected |
| parse(BufferedReader br, GenomicAnnotationFilter< T > filter, AnnotationHandler handler) | broad.core.annotation.AnnotationReader< T extends GenomicAnnotation > |  |
| parse(BufferedReader br, AnnotationFactory<?extends T > factory, GenomicAnnotationFilter< T > filter, AnnotationHandler handler) | broad.core.annotation.AnnotationReader< T extends GenomicAnnotation > | protected |
| permuteAnnotationsOnChromosome(final Map< String, Integer > sizes) | broad.core.annotation.AnnotationReader< T extends GenomicAnnotation > |  |
| shift(int amountToShift) | broad.core.annotation.AnnotationReader< T extends GenomicAnnotation > |  |
| shuffleScores(String chr, int setNum) | broad.core.annotation.AnnotationReader< T extends GenomicAnnotation > |  |
| shuffleScores(String chr) | broad.core.annotation.AnnotationReader< T extends GenomicAnnotation > |  |
| size() | broad.core.annotation.AnnotationReader< T extends GenomicAnnotation > |  |
| startTrack(String line) | broad.core.annotation.AnnotationReader< T extends GenomicAnnotation > |  |
| startTrack(String line, int trackNum) | broad.core.annotation.AnnotationReader< T extends GenomicAnnotation > |  |
| takeComplement(List<?extends Annotation > annotations) | broad.core.annotation.AnnotationReader< T extends GenomicAnnotation > |  |
| takeIntersection(List<?extends GenomicAnnotation > annotations) | broad.core.annotation.AnnotationReader< T extends GenomicAnnotation > |  |
| toStringForWriting() | broad.core.annotation.AnnotationReader< T extends GenomicAnnotation > |  |
| write(BufferedWriter bw) | broad.core.annotation.AnnotationReader< T extends GenomicAnnotation > |  |
| write(String outputFile) | broad.core.annotation.AnnotationReader< T extends GenomicAnnotation > |  |


---

Generated on Fri Aug 15 2014 15:10:50 for ESAT by  

 1.8.7
